# Novel Approach for Enterocutaneous Fistula Treatment with the Use of Viable Cryopreserved Placental Membrane

**DOI:** 10.1155/2016/8797691

**Published:** 2016-10-26

**Authors:** Frederick Nichols, Aaron Overly

**Affiliations:** Macomb Surgical Specialists, PLC, 27472 Schoenherr Road, Suite 150, Warren, MI 48088, USA

## Abstract

Enterocutaneous fistulas (ECF) are a difficult and costly surgical complication to manage. The standard treatment of* nil per os* (NPO) and total paraenteral nutrition (TPN) is not well tolerated by patients. TPN is also known for complications associated with long term central venous catheterization and for high cost of prolonged hospital stay. We present two low output ECF cases successfully treated with viable cryopreserved placental membrane (vCPM) placed into the fistula tracts. One patient is a 59-year-old male with a low output ECF from a jejunostomy tube site four weeks after the surgery. The second patient is an 87-year-old male with a low output ECF following a small bowel resection secondary to a strangulated inguinal hernia. He was evaluated on day 41 after surgery. NPO and TPN for several weeks did not resolute the ECF. The fistulae were closed postoperatively in both patients with zero output on the same day after one vCPM application. On day 3 postoperatively both patients were started on clear liquid diets and subsequently advanced to regular diets. The ECF have remained resolved for over 2 months. The use of vCPM is a novel promising approach for treatment of ECF.

## 1. Introduction

The treatment of ECF remains a challenging surgical problem. ECF result in malnutrition, sepsis, electrolyte, and fluid balance abnormalities, which are associated with considerable morbidity and mortality. A recent case series suggests a mortality rate of 6 to 33% associated with ECF [1]. The current standard of treatment for low output ECF is a conservative management, which usually lasts for months with recent reviews concluding that this can only resolve 30–60% of the fistula [2, 3]. The unresolved ECF require surgical management for closure following months of conservative management. Historically, other treatment modalities have been attempted; however most have been found to have unsatisfactory results [2, 4].

The use of placental membranes in the management of wounds and in surgical settings has been documented in the literature. For example, the amnion has been used for treatment of burns [5, 6], chronic leg wounds of various etiologies [7–9], ophthalmology [10], oral [11, 12] and ear [13] surgeries, reconstruction of pelvic floor [14], and vaginal epithelialization [15]. There are two placental membranes: the amniotic and the chorionic [16]. The amniotic membrane is composed of a single-cell epithelial layer and the stromal layer that contains fibroblasts and mesenchymal stem cells (MSCs) embedded in a collagen matrix. A loose spongy layer between the amniotic and chorionic membranes allows the amnion to be easily separated from the rest of the placenta and was most commonly used in the past. The chorionic membrane is composed of the mesenchymal layer, which like the amniotic membrane contains fibroblasts and MSCs in a collagen-rich matrix, and the chorionic trophoblast [17–19]. The main difference between the two placental membranes is higher tensile strength of the amniotic membrane attributed to the presence of the epithelial layer and the basement membrane [20]. The composition (collagen-rich extracellular matrix and endogenous growth factors and cells) and properties (anti-inflammatory, antibacterial, and antifibrotic) of placental membranes are supportive for tissue repair and regeneration [17, 20, 21]. Low immunogenicity of placental membranes allows the use of tissue allografts [22, 23]. Despite benefits of placental membranes, the use of fresh tissue has been limited due to its short shelf life and the risk of disease transmission. Progress in tissue preservation led to commercialization of placental membranes. Majority of commercial methods involve dehydration resulting in products that retain placental matrix and growth factors, but destroy tissue viable cells [20]. However, viable cryopreserved placental membranes that in addition to placental matrix and growth factors retain tissue viable cells are also available [20, 24]. Recently a use of a dehydrated amniotic allograft for surgical treatment of a vesicovaginal fistula has been reported [25]. The vesicovaginal fistula has been repaired using the da Vinci Surgical System with the placement of the amniotic membrane as an interposition patch over the repair [25]. The use as a patch is the most common for the placental membranes; however, for ECF with a narrow aperture an application of a dehydrated sheet of the amniotic membrane in the fistula is challenging.

## 2. Presentation of Cases

Grafix® (Osiris Therapeutics, Inc., Columbia, MD) is a human tissue allograft intended for the repair, replacement, or reconstruction of inadequate or damaged integumental tissue. Grafix is regulated by the FDA under 21 CFR Part 1271 Part 361 Human Cells, Tissues and Cellular and Tissue-Based Products (HCT/Ps) [24]. It is a cryopreserved placental membrane, in which the structural and cellular integrity of fresh placental membranes are preserved through a proprietary cryopreservation process. The cryopreservation involves the use of a dimethyl sulfoxide containing cryoprotectant solution at a controlled cooling rate [26]. Grafix is available in a form of amniotic (Grafix_PRIME_®) and chorionic (Grafix_CORE_®) membranes. Extensive donor screening and serological, bioburden, and sterility testing are performed on every lot to demonstrate suitability for clinical use. Each lot is tested to confirm cellular viability postthaw [24].

*Case  1*. A 59-year-old male who presented to the emergency room in septic shock with free air on radiologic imaging was resuscitated, intubated, and brought to the operating room for exploratory laparotomy. Past medical history was only positive for hyperlipidemia, and he has no past surgical history. Upon exploratory laparotomy the patient was found to have a perforated jejunal ulcer, which was biopsied, and then oversewn using 3-0 silk and nearby mesentery as a buttress. A jejunal feeding tube was placed distal to the ulcer repair. Postoperatively, the patient course developed a complication secondary to severe sepsis and multisystem organ failure. He underwent bilateral below the knee amputations due to ischemia related to vasopressor support on post-op day 9 and was placed on hemodialysis. On post-op day 14 the patient was tolerating a regular diet and the jejunal feeding tube was removed. He subsequently developed an ECF with 2 mm aperture at the feeding tube site. He was made NPO and started on TPN and somatostatin. The fistula was low output, producing an average of 60–120 mL per day. On post-op day 27 the patient was taken to the operating room for application of vCPM into the fistula tract using a lacrimal duct probe to introduce the placental membrane sheet into the tract. On day 1 after application of vCPM he was kept NPO on TPN and had zero output from his fistula. On day 3 after the vCPM application the patient was started on a clear liquid diet and advanced to regular diet on day 6. He was discharged to a rehabilitation center where he has been followed up. Two months later he has continued to have zero output from the fistula. 

*Case  2*. An 87-year-old male presented to the emergency room with an incarcerated left inguinal hernia. He has a past medical history of atrial fibrillation, coronary artery disease, and hypertension. He has a past surgical history of coronary artery bypass grafting and repair of perforated peptic ulcer. He was taken to the operating room. He was found to have a strangulated and gangrenous segment of small bowel in his scrotum. He underwent a small bowel resection with primary anastomosis along with orchiectomy and hernia repair. He subsequently developed an ECF with 2 mm aperture treated conservatively with NPO, TPN, and somatostatin without success. The patient was having low output of 10–20 mL per day, and on post-op day 40 the patient was taken to the operating room for application of vCPM into the fistula tract. Using a 14-gauge angiocatheter on a 3-cubic-centimeter syringe with 1 cubic centimeter of saline as a carrier fluid the membrane was then injected into the fistula tract while the syringe was slowly withdrawn leaving approximately 10% of the membrane on the superficial surface. The patient was kept NPO on TPN after vCPM application and had zero output from the fistula. On day 3 of vCPM application he was started on a clear liquid diet and advanced to a full liquid diet on day 6. Two months later the patient has had zero output from the fistula.

## 3. Discussion

Placental membranes have anti-inflammatory, antimicrobial, antifibrotic, and angiogenic properties [17, 20, 21]. These properties of placental membranes attributed to their unique composition: a collagen-rich extracellular matrix, growth factors, and endogenous viable cells, including epithelial cells, fibroblasts, and mesenchymal stem cells [17–19]. Current progress in tissue preservation resulted in commercialization of placental membranes. Grafix, a viable cryopreserved placental membrane (Osiris Therapeutics, Inc.), is a commercial product that retains all components of placental tissue in their native state [24]. The cryopreservation of Grafix involves the use of a dimethyl sulfoxide containing cryoprotectant solution at a controlled cooling rate, according to a proprietary process developed by Osiris Therapeutics, Inc. [26]. Such method results in higher than 70% cell viability of the tissue postthaw [26]. Scientific reports show that preservation of all components of placental membranes in their native state is required to retain all properties of fresh tissue [26–28]. Retrospective and prospective clinical studies have demonstrated benefits of Grafix Prime for wounds of various etiology including venous stasis ulcers, diabetic foot ulcers, pressure ulcers, and surgical dehiscence [29, 30]. The clinical effectiveness of viable cryopreserved chorionic membrane for complex wounds with exposed tendon and/or bone has been demonstrated in a recent prospective multicenter clinical trial [31]. In this study 96.3% patients achieved 100% granulation of the wound bed, and 59.3% of patients completely closed their wounds [31]. Because ECF is a surgical wound and placental membranes are beneficial for wounds including surgical ones [32], we hypothesized that placental membranes might be beneficial for ECF management. One literature report supports our hypothesis. In this case study of the dehydrated amnion has been used to treat a vesicovaginal fistula [25]. The amniotic membrane has been applied as an interposition patch over surgically repaired fistula [25]. In our study we used the viable cryopreserved chorionic membrane. Selection of this product was based on better preservation of all components of fresh placental tissue and handling properties of the chorionic membrane. The chorionic membrane is jelly-like that allows easy application into a narrow fistula's tract. Our hypothesis is that the soft chorionic graft fills fistula and supports formation of granulation tissue followed by the closure of the fistula similar as it was reported for complex leg wounds [31].

We present here two low output ECF cases that were refractory to the conservative treatment. Both patients received an application of vCPM into the fistula tract. Both patients had zero output on the same day of vCPM placement. Both patients had resolution of their ECF after receiving treatment with the vCPM and were able to maintain a regular diet without recurrence to date. We believe that vCPM is a promising treatment modality for low output ECF in addition to the standard conservative management and may allow for a significant reduction of the duration of treatment. In addition, this would allow for a concomitant reduction in the cost of TPN administration, length of stay in the hospital, and potential complications related to the need for maintaining central venous catheterization and associated care. There have been no published reports in the literature describing such technique being used for treatment of ECF. Results of the presented cases show potential benefits of vCPM for low output ECF. Further studies including a controlled clinical trial are required to confirm our initial promising results.
